# Basic and Clinical Advances in Chronic Liver Inflammation

**DOI:** 10.1155/2016/1571457

**Published:** 2016-02-24

**Authors:** Hirayuki Enomoto, Akihiro Tamori, Hitoshi Yoshiji, Ekihiro Seki

**Affiliations:** ^1^Division of Hepatobiliary and Pancreatic Disease, Department of Internal Medicine, Hyogo College of Medicine, Mukogawa-cho 1-1, Nishinomiya, Hyogo 663-8501, Japan; ^2^Department of Hepatology, Osaka City University Graduate School of Medicine, 1-4-3 Asahimachi, Abeno-ku, Osaka 545-8585, Japan; ^3^Third Department of Internal Medicine, Nara Medical University, Shijo-cho 840, Kashihara, Nara 634-8521, Japan; ^4^Division of Gastroenterology, Department of Medicine, Cedars-Sinai Medical Center, 8700 Beverly Boulevard, Davis, Suite D2099, Los Angeles, CA 90048, USA

Continuous liver inflammation causes fibrotic changes and leads to the development of liver cirrhosis and liver cancer. Recent biological and medical advances have clarified the mechanisms of chronic liver inflammation and succeeded in providing new therapies for various liver diseases. We proposed this special issue to provide recent basic and clinical findings in chronic liver inflammation and its complications.

Regarding the diagnosis and treatment of liver inflammation, A. Tsutsui et al. showed the clinical utility of the Digestive Disease Week Japan 2004 (DDW-J) scale, which has been used as an objective diagnostic tool for drug-induced liver injury in Japan. A. Tamori et al. reviewed remarkable progression in antiviral treatments for hepatitis C virus (HCV), including DAAs (direct-acting antivirals or direct antiviral agents).

We also introduce papers regarding the complications of progressed liver diseases in this special issue. The prognoses of cirrhotic patients are highly dependent on their liver function, and A. Hassan et al. showed that L-carnitine administration helps maintain and improve liver functions after transarterial chemoembolization. Additionally, malnutrition is a frequently observed complication which is known to be associated with a poor prognosis. Y. Osaki and H. Nishikawa reviewed the nutritional problems of cirrhosis, focusing on a recent hot topic “sarcopenia.” Portal hypertension is a major problem along with the progression of chronic liver disease. The paper by K. Kotani et al. suggested the association of the immune system with the development of portal hypertension. Hepatocellular carcinoma is also a prognosis-determining complication of patients with chronic liver inflammation. K. Shindo et al. showed the clinical utility of a semiannual imaging surveillance program in patients without hepatitis viral infection.

With regard to the basic mechanisms of chronic liver inflammation, H. Tsutsui et al. reviewed the roles of IL-1 family cytokines in the development of various liver diseases, including IL-1 family cytokine-mediated molecular and cellular networks. Coinfection of HCV and human immunodeficiency virus (HIV) cooperatively leads to the progression of liver disease. Along this viewpoint, the paper by M. C. Liberto et al., which described the specific cross talk among HIV and HCV proteins in coinfected patients, is interesting and clinically relevant.

We hope that these papers will provide new findings for basic and clinical researchers and help promote their studies for the development of better management for patients with chronic liver inflammation.

